# Enhanced Stability and Oral Bioavailability of Cannabidiol in Zein and Whey Protein Composite Nanoparticles by a Modified Anti-Solvent Approach

**DOI:** 10.3390/foods11030376

**Published:** 2022-01-27

**Authors:** Ce Wang, Jia Wang, Yonghai Sun, Kalev Freeman, Monique Alyssa Mchenry, Cuina Wang, Mingruo Guo

**Affiliations:** 1 Department of Food Science, College of Food Science and Engineering, Jilin University, Changchun 130062, China; wangce18@mails.jlu.edu.cn (C.W.); wangjia9919@mails.jlu.edu.cn (J.W.); sunyh@jlu.edu.cn (Y.S.); 2 College of Medicine, University of Vermont, Burlington, VT 05405, USA; Kalev.Freeman@uvm.edu (K.F.); Monique.Mchenry@uvm.edu (M.A.M.); 3 Department of Nutrition and Food Sciences, College of Agriculture and Life Sciences, University of Vermont, Burlington, VT 05405, USA; 4 Dairy Science Laboratory, Northeast Agricultural University, Harbin 150030, China

**Keywords:** cannabidiol, zein, whey protein, nanoparticle

## Abstract

Wide applications of cannabidiol (CBD) in the food and pharmaceutical industries are limited due to its low bioavailability, sensitivity to environmental pressures and low water solubility. Zein nanoparticles were stabilized by whey protein (WP) for the delivery of cannabidiol (CBD) using a modified anti-solvent approach. Particle size, surface charge, encapsulation efficiency, and re-dispersibility of nanoparticles were influenced by the zein to WP ratio. Under optimized conditions at 1:4, zein–WP nanoparticles were fabricated with CBD (200 μg/mL) and further characterized. WP absorbed on zein surface via hydrogen bond, hydrophobic forces, and electrostatic attraction. The zein–WP nanoparticles showed excellent storage stability (4 °C, dark) and effectively protected CBD degradation against heat and UV light. In vivo pharmacokinetic study demonstrated that CBD in zein–WP nanoparticles displayed 2-times and 1.75-fold enhancement in maximum concentration (C max) and the area under curve (AUC) as compared to free-form CBD. The data indicated the feasibility of developing zein–WP based nanoparticles for the encapsulation, protection, and delivery of CBD.

## 1. Introduction

Cannabidiol (CBD), a major non-psychotropic constituent of the Cannabis sativa plant, has biological activities such as anti-convulsive, anti-anxiety, anti-psychotic activity [1]. However, wide applications of CBD in food and pharmaceutical industries are hindered by variable pharmacokinetic profiles caused by its inherent attributes, such as low water solubility, sensitivity to environmental pressures, and low bioavailability [2]. CBD is highly lipophilic (log P = 6.3) and, thus, it is commonly incorporated in alcohol-based formulations or oil [2,3]. CBD may experience isomerization, polymerization, or degradation when exposed to harsh environments, e.g., heating, light, and oxygen [2]. Oral administration of CBD is challenging, with a bioavailability of approximately 6% in humans [4] due to poor solubility in the gastrointestinal system [5]. It has been comprehensively understood that the fabrication of delivery systems may conquer these hindrances.

In recent years, a few delivery systems have been constructed to encapsulate CBD, including nano-emulsions [4,6], Pickering emulsions [7], and inclusion complexes [8]. However, emulsions have some disadvantages. First, emulsifiers are required for producing stable emulsions, and some chemically synthetic surfactants are usually toxic and harmful to humans. Second, high-energy input emulsifying devices such as ultrasonic or high-pressure homogenization equipment are usually needed to reduce the droplet size. Third, the emulsification process is usually complicated, containing several steps. Inclusion complexes usually result in weak binding of ligands with vectors. Hereby, we focused on developing a natural biopolymer-based nanoparticle delivery systems using a simple method.

Zein is a byproduct of producing corn starch and exhibits strong hydrophobic characteristics. The exceptional self-assembly capability of zein to create nanoparticles makes it an advantageous delivery carrier for nutraceuticals [9]. Entrapment in zein nanoparticles could substantially enhance the stability and bioavailability of guest compounds [9]. However, the constructed particles still bear high hydrophobicity and readily aggregate via hydrophobic interaction. Whey protein (WP), isolated from bovine milk, is mainly composed of β-lactoglobulin (70%) along with other proteins (α-lactalbumin, lactoferrin, bovine serum albumin). WP was capable of absorbing on zein nanoparticles via generating an interpolymer complex as a result of its amphiphilic and charged nature, and could prevent colloid aggregation [9]. The consequent zein–whey protein core–shell nanoparticles were documented to remarkably enhance the solubility, re-dispersibility, stability, and oral bioavailability of encapsulated active substances in comparison with pure zein [10,11,12]. 

The most employed technique to fabricate zein-based nanoparticles is anti-solvent precipitation (ASP) [11]. Traditionally, ASP is used to introduce organic zein solution drop by drop into an antisolvent, which is time-consuming and difficult to scale up in practical application. To solve this problem, a modified easy-operating anti-solvent procedure was developed by directly pouring the aqueous solution into an organic zein solution [13,14] which can also encapsulate bioactive ingredients into the core. Herein, a CBD loaded zein–whey protein composite delivery system was fabricated using the modified ASP and characterized for its physicochemical properties, stability, and bioavailability.

## 2. Materials and Methods

### 2.1. Materials 

Zein (purity of 92%) was purchased from Yuanye Bio-Technology Co., Ltd. (Shanghai, China). Whey protein with a purity of 93.14% was obtained from Fonterra Co-operative Group (Auckland, New Zealand). Cannabidiol (CBD, purity of 99%) was purchased from Macklin Biochemical Co., Ltd. (Shanghai, China). Potassium bromide was purchased from Sigma-Aldrich (St. Louis, MO, USA). 

### 2.2. Fabrication of CBD-Loaded Composite Nanoparticles

**Figure 1:**
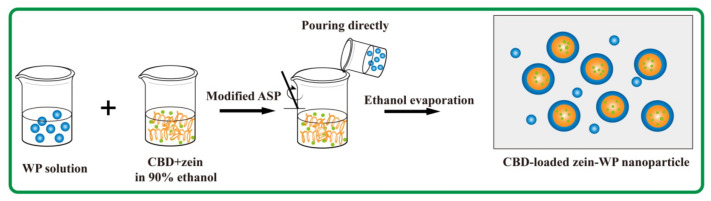
Synthesis of CBD-loaded zein–WP nanoparticles.

CBD-loaded zein–WP composite nanoparticles were fabricated based on a modified ASP described in a previous study [13], and the process was as depicted in Figure 1. Briefly, CBD powder was dissolved in zein solution (25 mg/mL, 90% ethanol) at a final concentration of 1 mg/mL. WP solutions (pH 7) at various concentrations were rapidly poured into CBD-containing zein solution within 1 s under continuous stirring (1000 rpm) using a magnetic stirrer (IKA, Staufen, Germany). Ethanol in nanoparticle suspension was removed by vacuum rotatory evaporator at a 40 °C water bath, and distilled water at a volume equal to that of the lost ethanol was added. Final nanoparticle suspensions were obtained by centrifuging at 2000 rpm for 10 min to remove large precipitates. The final mass ratios of zein to WP were 1:0, 1:2, 1:3, 1:4, and 1:5. 

### 2.3. Particle Size and Zeta-Potential 

The size distribution by intensity and zeta-potential of samples were estimated using dynamic light scattering and phase analysis light scattering techniques with a Nano Zetasizer (Malvern Instruments Ltd., Worcestershire, UK) equipped with a He-NE laser (633 nm). Zein–WP composite nanoparticle dispersions were diluted by 10 folds with Milli-Q water before measurement. 

### 2.4. High Performance Liquid Chromatography (HPLC)

HPLC analysis of CBD was conducted using a Waters system equipped with a C_18_ column (5 μm, 4.6 mm × 150 mm) (Waters Corporation, Milford, DE, USA), according to a previous study [15], with some modifications. CBD was separated using a mobile phase of water (A) and acetonitrile (B) at a flow rate of 0.5 mL/min. The gradient was as follows: 0–13 min, 70–85% B; 13–13.1 min, 85–95% B; 13.1–14 min, isocratic elution with 95% B; 14–14.1 min, 95–85% B; 14.1–17 min, 85–70% B; 17–19 min, isocratic elution with 70% B. The injection volume was 10 µL, and the column temperature was 25 °C. Samples were detected at a wavelength of 228 nm and the CBD was quantified based on an established standard curve (R^2^ > 0.999). 

### 2.5. Encapsulation Efficiency (EE) and Loading Capacity (LC)

The nanoparticle suspension was mixed with acetonitrile at 9-folds volume, vortexed for 2 min, and then centrifuged at 14,000× *g* for 20 min. The supernatant was determined for CBD content (C_total_) which contained encapsulated and free CBD. For determination of free CBD, the nanoparticle suspension was first centrifuged at 14,000× *g* for 20 min twice to precipitate the nanoparticles, and then the supernatant was mixed with acetonitrile. The supernatant was assayed for CBD content (C_free_). EE and LC were calculated using the following equations:

(1) $$\mathrm{EE}\;(\%)=\frac{C_{\mathrm{total}}-C_{\mathrm{free}}}{C_{\mathrm{initial}}}\;\times \;100$$

(2) $$\mathrm{LC}\;(\%)=\frac{C_{\mathrm{total}}-C_{\mathrm{free}}}{\mathrm{weight}\;\mathrm{of}\;\mathrm{zein}\;\mathrm{and}\;\mathrm{WP}\;\mathrm{input}}\;\times \;100$$

where C_initial_ is the initial concentration of CBD added into the system, and C_total_ and C_free_ are the concentration of total and free CBD in the colloid system, respectively. 

### 2.6. Water Solubility 

The water solubility of CBD in the nanoparticle suspensions was determined and compared to that of CBD alone. Pure CBD powder at the equivalent amount to that in nanoparticles was dissolved in 10 mL distilled water and magnetically stirred at 100 rpm for 2 h. The sample was filtered by a 0.22 µm Millipore filter to remove insoluble CBD. CBD in the nanodispersion was extracted following the method described for obtaining total CBD in Section 2.5, and the concentration was determined by HPLC as described in Section 2.4. The water solubility of CBD was calculated using the following formula:

(3) $$\mathrm{Water}\;\mathrm{solubility}\;(\mu g/\mathrm{mL})=\frac{C_{\mathrm{total}}}{\mathrm{solvent}\;\mathrm{volume}}$$

### 2.7. Re-Dispersibility of Freeze-Dried Nanoparticles

CBD-loaded nanoparticle suspensions were frozen at −20 °C overnight and then lyophilized using a freeze drier (ALPHA 1–2, CHRIST, Osterode, Germany). Lyophilized CBD-loaded nanoparticles were re-dispersed in Milli-Q deionized water to original volumes and then stirred for at least 1 h. Redissolved samples were observed for appearance by taking pictures and assessed for physical properties including particle size, PDI, zeta-potential, and CBD re-dispersibility rate. CBD in nanodispersion was extracted following the method described for obtaining total CBD in Section 2.5 and the concentration was determined by HPLC as described in Section 2.4.

(4) Re-dispersibility of CBD (%)= Cre-dispersedCfresh × 100

where C_re-dispersed_ is the concentration of CBD in the re-dispersed system and C_fresh_ is the concentration of CBD in the fresh colloid system. 

### 2.8. X-ray Diffraction (XRD)

X-ray diffractograms of CBD, zein, WP, the physical mixture of two biopolymers with CBD, and freeze-dried CBD-loaded nanoparticles were recorded using an XRD diffractometer (MM007HF/R-AXIS RAPID II, Rigaku Industrial Corporation, Osaka, Japan). Data were acquired in the angular range of 2θ = 5–60° and scanning steps of 0.02°.

### 2.9. Fourier Transform Infrared Spectrometry (FT-IR) 

FT-IR spectra of CBD, zein, WP, the physical mixture of two biopolymers with CBD, and freeze-dried CBD-loaded nanoparticles were obtained using an IRPRESTIGE-2 FT-IR spectrometer (Shimadzu, Tokyo, Japan). Samples (2 mg) were mixed with pre-dried KBr (200 mg) and then pressed into tablets. FT-IR spectra data were collected in the range of 500–4000 cm^−1^ at a resolution of 4 cm^−1^.

### 2.10. Transmission Electron Microscopy (TEM) 

The morphology of CBD-loaded nanoparticles at zein to WP ratio of 1:4 was imaged using a Transmission Electron Microscope (H-7650, HITACHI, Tokyo, Japan) at 100 kV, with a magnification of 7000. Fresh dispersions were diluted using distilled water and stained with uranyl acetate. Representative TEM images were reported.

### 2.11. Stability 

The physicochemical stability and storage stability of nanoparticle dispersions were evaluated. For thermal stability testing, fresh nanoparticle suspensions were heated at 80 °C for 10, 30, 60, and 90 min in a water bath, and then cooled down to 25 °C quickly. Samples were assayed for particle size and CBD retention rate. Fresh CBD nanoparticles and pure CBD solution (20% ethanol) were irradiated by UV light for 15, 30, 45, 60, 75, and 90 min. The CBD retention rate in each sample was calculated. For storage stability, fresh CBD nanoparticles were stored at 4 °C in the dark for 21 days, followed by measurement for CBD retention rate and particle size. CBD in the nanodispersion was extracted following the method described for obtaining total CBD in Section 2.5, and the concentration was determined by HPLC as described in Section 2.4.

(5) $$\mathrm{Retention}\;\mathrm{rate}\;\mathrm{of}\;\mathrm{CBD}\;(\%)=\frac{{\;C}_{\mathrm{residual}}}{C_{\mathrm{initial}}}\;\times \;100$$

where C_residual_ is the concentration of CBD in the system after exposure to environmental stresses, and C_initial_ is the initial concentration of CBD added into the system.

### 2.12. In Vivo Bioavailability Study

Male Sprague Dawley rats (mean weight of 300 ± 15 g) at the Specific Pathogen Free grade were provided by Beijing HFK Bioscience Co., Ltd. (Beijing, China). All animals were housed in plastic animal cages in a ventilated room, where they were maintained at 20–26 °C and 40–60% relative humidity with a 12-h light/dark cycle. Water and commercial laboratory complete food were available ad libitum, and animals were acclimated to environment for 7 days before the experiment. The animal studies complied with the guidelines of Jilin University on animal care (Number of permit: SY202110009).

Rats were randomized into two groups and fasted overnight before oral administration. Zein–WP nanoparticle and pure CBD with an equal CBD amount at 40 mg/kg were orally administered to rats. After administration, blood was collected in heparin tubes at designed timepoints of 0, 0.5, 1, 1.5, 2, 4, 8, and 12 h by orbital puncture. Plasma was collected by centrifuging blood at 6000 rpm for 10 min and then stored at −20 °C until analysis. 

For extracting CBD, plasma samples were mixed with methanol at 3-fold volume and 5-times n-hexane, and then vortexed for 5 min. The organic layer was then obtained by centrifugation at 9000× *g* for 10 min and dried by nitrogen stream. The residue was redissolved using acetonitrile and then subjected to HPLC analysis. Pharmacokinetic parameters including maximum concentration (C_max_), time to maximum concentration (T_max_), area under the concentration-time curve (AUC), and mean residence time (MRT) were estimated by DAS 2.0 (BioGuider Co., Shanghai, China). 

### 2.13. Statistical Analysis

Data are the average value of triplicates of at least three batches and expressed as mean ± SD. Statistical analysis of data was conducted using SPSS version 21 (SPSS Inc., Chicago, IL, USA). One-way analysis of variance followed by a Least Squared Differences (LSD) model were used to compare groups at significance level of 0.05.

## 3. Results and Discussion

### 3.1. Particle Size and Zeta-Potential

**Figure 2:**
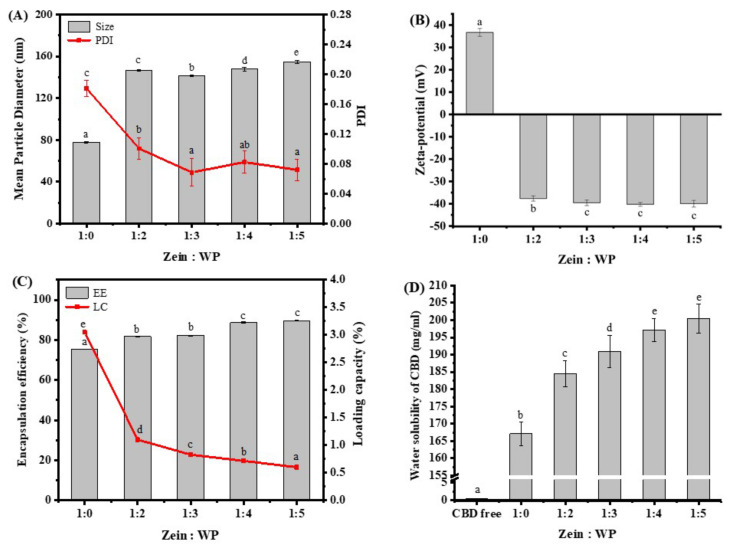
Mean particle diameter, polydispersity index (PDI) (**A**), and zeta-potential (**B**) of nanoparticles at zein:WP ratios of 1:0 to 1:5. Encapsulation efficiency (EE), loading capacity (LC) (**C**), and water solubility (**D**) of CBD in nanoparticles at zein:WP ratios of 1:2 to 1:5. Note: Different lowercase letters indicate the significant difference (*p* < 0.05) between samples at different biopolymer ratios.

The mean particle size and polydispersity index (PDI) of nanoparticles fabricated at different zein:WP ratios are exhibited in Figure 2A. CBD-loaded zein nanoparticles (CBD/zein) displayed an average particle size of 78 nm. The particle size was significantly increased in CBD/zein–WP by comparison with CBD/zein (*p* < 0.05), attributed to the fact that WP was absorbed in the core due to the hydrophobic effect and electrostatic interaction. In addition, a larger core of zein may be formed when directly added into WP solution. With increasing WP concentration, the particle size of composite nanoparticles significantly decreased with changing zein:WP ratios from 1:2 to 1:3, and then increased (1:4 and 1:5), *p* < 0.05. The decrease may be due to electrostatic repulsion and steric hindrance [16], while the increase was due to the more involved interference of WP with particle formation and a heavy coating of WP on the surface of zein nanoparticles [17]. 

Notably, the size of composite nanoparticles was still small, in the range of 140–160 nm, even though they are increased compared with the that of zein particle. The small particle size may be due to the fact that directly pouring WP solution into the zein ethanol/water binary solvent resulted in immediate access of sufficient WP on the surface of zein nanoparticles after it began to aggregate, avoiding the occurrence of larger particles due to steric hindrance and the interface disturbance effect [13]. More importantly, small particle size is a key factor for improving the oral performance of drugs when incorporated into nanoparticles. Small particle size means large surface area and saturation solubility, which in turn improve the release rate of the drug and provides a high concentration in the gastrointestinal tract [18]. 

As demonstrated in Figure 2A, the PDI of a nanoparticle with sole zein as an encapsulating material was approximately 0.18, and the magnitude was significantly reduced to 0.06–0.1 upon introduction into the WP solution (*p* < 0.05), revealing that nanoparticles were uniform colloidal systems [19]. This result may have been because adequate WP molecules can approach the surface of zein aggregates promptly and become well allocated on particles throughout the anti-solvent procedure [13]. Specifically, when quickly mixing anti-solvent with CBD-containing zein ethanol aqueous solution, one brief outburst of nuclei and a high nucleus concentration would occur. Subsequently, the resulting nuclei will develop evenly by capturing dissolved zein molecules [20], producing narrowly distributed small particles.

Figure 2B illustrates that zein nanoparticles were positively charged (36.70 ± 1.76 mV), while the combination with WP led to nanoparticle zeta-potential conversion, implying that negatively charged WP molecules were attached on the exterior of the zein. The data also testified to the occurrence of electrostatic interaction between zein and WP. Our findings also demonstrated that the zeta-potential of samples with changing zein:WP proportions climbed significantly (*p* < 0.05), and then remained at a permanently elevated level. The constant surface charge of composite nanoparticles may have been associated with the progressively saturated interaction between zein and WP when the ratio reached 1:3 [21]. Concurrently, it should be noticed that great zeta-potential values (about −40 mV) of all samples revealed that nanoparticles could be stabilized via powerful electrostatic repulsion [10] which may be provided by negatively charged amino acids such as aspartic acid and glutamic acid.

### 3.2. Encapsulation Efficiency (EE), Loading Capacity (LC) and Water Solubility

Encapsulation efficiency (EE) and loading capacity (LC) are frequently used to assess whether the nanoparticle is an applicable delivery system [21]. Figure 2C demonstrates that zein nanoparticles displayed EE values at around 75% due to the intrinsic hydrophobicity of CBD, similar to a previous study showing that the EE for lutein encapsulated by zein-based nanoparticles can be up to 80% at a 25:1 mass ratio of zein to lutein [22]. In our case, the EE of fabricated composite nanoparticles was significantly increased to about 89% (*p* < 0.05) with nanoparticles at zein:WP ratios of 1:4 and 1:5 reaching the highest value. The remarkedly higher EE may be due to two reasons. First, the possible larger cavity of the zein core nanoparticles in the composite system could accommodate more CBD molecules. Additionally, free CBD may be embedded between the zein core and the WP shell and captured by redundant WP due to its amphiphilicity. These results denoted that there existed a synergistic effect between zein and WP in promoting the embedding ability of colloidal complex nanoparticles [23]. In terms of LC, nanoparticles at polymer ratio of 1:0 showed the significantly highest value (*p* < 0.05), due to the lower mass of polymers.

The solubility of pure CBD in water was measured to be around 0.39 µg/mL, which was higher than rate reported in previous studies, being 0.06 µg/mL [24] and 0.02 µg/mL [25]. Figure 2D indicates that CBD in zein nanoparticle had a solubility of 167 µg/mL, and the value was significantly improved to 184–200 µg/mL following encapsulation into composite nanoparticles (*p* < 0.05) due to their enhanced encapsulation efficiency. Clearly, a significant improvement (*p* < 0.05) of about 465, 481, 496, and 505 folds for water solubility of CBD was observed after encapsulation in zein–WP nanoparticles as the polymer ratio grew from 1:2 to 1:5, as compared with free CBD. The enhancement of the water solubility of phytochemicals is beneficial to incorporate phytochemicals into water-soluble foods and improve their bioavailability in vivo [26].

### 3.3. Re-Dispersibility of Freeze-Dried Composite Nanoparticles

**Figure 3:**
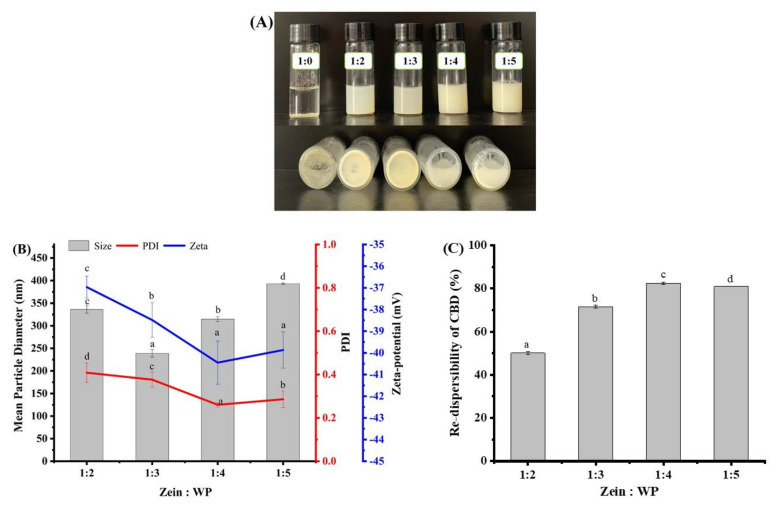
Re-dispersibility of freeze-dried CBD-loaded nanoparticles as demonstrated by nanoparticle appearance (**A**), mean particle diameter, PDI, zeta-potential (**B**), and re-dispersibility of CBD (**C**). Note: different lowercase letters indicate the significant difference (*p* < 0.05) between samples at different biopolymer ratio.

The dehydration and rehydration properties of particles are of great importance and can be investigated by evaluating their dispersibility in water after drying [27]. As displayed in Figure 3A, after freeze-drying, CBD/zein was no longer re-dispersible resulted from irreversible aggregation due to the strong hydrophobicity [28]. This unwanted phenomenon was alleviated in CBD/zein–WP, indicating that water solubility was enhanced by hydrophilic/amphiphilic WP shell coated on zein outer surface [28], which boosted the water-binding capacity of nanoparticles and provided electrostatic repulsion and steric stabilization [24]. Nevertheless, insoluble aggregates were still observed for dispersible lyophilized nanoparticles at zein:WP ratios of 1:2 and 1:3, denoting that WP molecules may be not enough to fully cover all zein particles.

The re-dispersed nanoparticles showed obviously increased average particle sizes compared with fresh nanoparticles (Figure 2A and Figure 3B). Nanoparticles at a zein:WP ratio of 1:2 had a larger particle size than samples at ratios of 1:3 and 1:4. This may be attributed to the partially exposed zein nanoparticle surface which aggregated during the lyophilization-rehydration process. In terms of PDI (Figure 3B), the most homogeneous suspension of nanoparticles was achieved when the ratio of zein to WP was 1:4. Additionally, the highest values of zeta-potential and re-dispersibility of CBD in nanoparticles were also achieved at a mass ratio of 1:4. Considering particle size, surface charge, EE, and re-dispersibility of nanoparticles, an optimized zein:WP ratio at 1:4 was chosen for further characterization. 

### 3.4. XRD Diffractogram 

**Figure 4:**
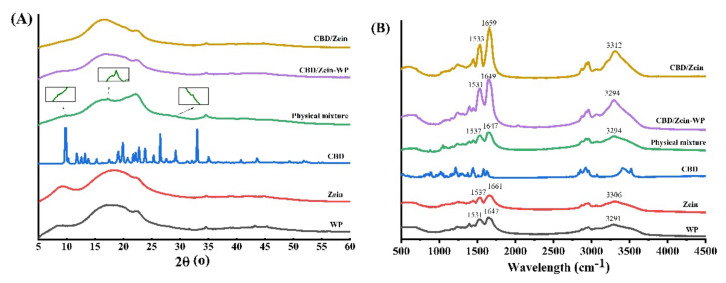
X-ray diffraction (XRD) patterns (**A**) and Fourier transform infrared (FT-IR) spectra (**B**) of WP, zein, CBD, a physical mixture (CBD, WP and zein), CBD-loaded zein nanoparticles (CBD/zein), and CBD-loaded zein–WP nanoparticles (CBD/zein–WP).

CBD encapsulated in nanoparticles at a zein:WP ratio of 1:4 was confirmed by XRD spectra (Figure 4A). Pure crystalline CBD exhibited characteristic peaks between 5° and 40° (2θ = 9.66°, 10.24°, 11.78°, 12.53°, 13.08°, 15.34°, 19.85°, 19.03°, 21.66°, 22.73°, 23.79°, 25.29°, 26.42°, 29.19°, 32.98°), similar to those found in a previous study [8]. Zein and WP were of amorphous form, and no sharp peaks were observed in the XRD diffractogram [29]. Physical mixtures containing the same ingredients at equivalent amounts showed similar peaks with individual CBD at smaller intensity (as labeled by arrows). Upon embedding in nanoparticles, the main typical peaks of CBD between 5° and 40° disappeared. The disappearance of the peaks for CBD after encapsulation may be due to the low amount incorporated. Additionally, by binding with protein molecules, the crystallization tendency of CBD was inhibited, forming amorphous complexes [30]. The amorphous state is favorable for CBD application in oral administration due to its higher oral bioavailability than its crystalline counterpart [28]. 

### 3.5. FT-IR Spectra 

As depicted in Figure 4B, individual CBD exhibited two significant characteristic bands at 3520 cm^−1^ and 3410 cm^−1^ corresponding to the stretching vibration of -OH, a peak at 3073 cm^−1^ assigned to the C-H stretching vibration of the benzene ring, peaks at 2963 cm^−1^, 2924 cm^−1^, 2855 cm^−1^, and 2830 cm^−1^ denoted for the stretching vibration of -CH_3_ and -CH_2_-, peaks at 1626 cm^−1^, 1582 cm^−1^, 1512 cm^−1^, and 1441 cm^−1^ corresponding to the benzene skeleton vibration, a peak at 1373 cm^−1^ denoted for the bending vibration of -CH_3_, and one at 1215 cm^−1^ for the C-O stretching vibration [25]. The FT-IR spectra of physical mixtures were presented as a simple superposition of CBD, WP, and zein, suggesting no interaction occurred when they were mixed physically [25]. However, characteristic peaks of CBD disappeared or merged in the spectra of the nanoparticles due to the limitation of stretching and bending of vibrations in the CBD molecule when bound to protein [31]. Similar results were reported for resveratrol after incorporation into composite particles [28]. Additionally, the low CBD concentration may also affect its FT-IR signal.

Zein showed three main typical peaks at 3306, 1661, and 1537 cm^−1^, which were ascribed to the stretching vibration of hydroxy groups, C=O stretching (amide I), and stretching of C-N coupled with the bending of N-H (amide II), respectively. Compared with zein, the peak of -OH groups in nanoparticles (CBD/zein) shifted to 3308 cm^−1^ and 3312 cm^−1^, revealing that hydrogen bonding occurred between zein and CBD. The presence of CBD also changed two major characteristic peaks of zein to 1659 cm^−1^, 1659 cm^−1^ (amide I), and 1535 cm^−1^, 1533 cm^−1^ (amide II), respectively, and their peak intensity was significantly increased. The findings demonstrated that electrostatic and hydrophobic interactions might exist between zein and CBD, which were ascribed to amide groups of glutamines in zein and hydroxyl and carbonyl groups in CBD [32]. 

When zein formed composite nanoparticles with WP, stretching vibration peak intensity of -OH increased and a blue shift (3294 cm^−1^) occurred. This suggested that intermolecular forces between zein and WP included not only electrostatic attraction due to their opposite charges but also hydrogen bonding. Similarly, a previous study reported that hydrogen bonding was one of the dominant driving forces in the formation of zein and WP nanoparticles [10]. Compared with the spectrum of CBD/zein, little change of peak in amide II was observed in CBD/zein–WP, while amide I was significantly shifted. Changes in the amide I band possibly resulted from the electrostatic and hydrophobic interaction between zein and WP in the particles [33]. Therefore, WP molecules may bind to the surface of zein particles by electrostatic force, hydrophobic force, and hydrogen bond, forming a core–shell structure. 

### 3.6. Microstructure Observed by TEM 

**Figure 5:**
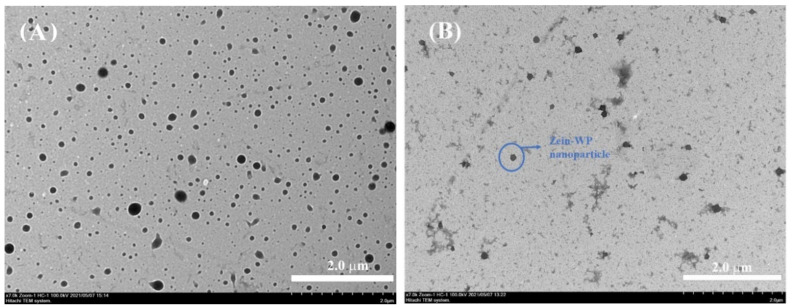
TEM images of CBD-loaded zein nanoparticle (**A**) and CBD-loaded zein–WP nanoparticle (**B**).

The microstructure of nanoparticles is shown in Figure 5. The size distribution in TEM photographs was in accordance with the results from dynamic light scattering (Figure 2A), where the mean particle size of zein nanoparticles was smaller than that of zein–WP nanoparticles. Uniformly smoothly spherical shaped nanoparticles were observed for both samples. When anti-solvent was rapidly mixed with zein dispersion, a 20% ethanol-water binary solvent system was formed immediately, which may have led to stable zein nanoparticles with an ordered structure. 

### 3.7. Physicochemical Stability

**Figure 6:**
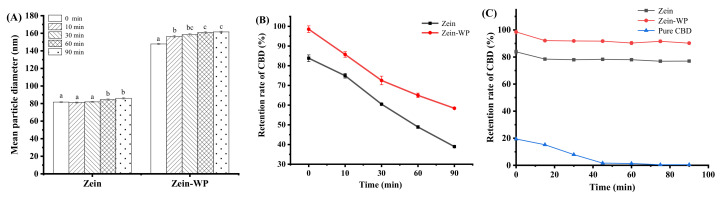
Effects of environmental stress on properties of nano-dispersions. (**A**,**B**) The mean particle diameter and CBD retention rate of nano-dispersions after heating at 80 °C for 0–90 min; and (**C**) the retention rate of CBD in ano-dispersions and pure CBD under UV light irradiation for 90 min. Note: different lowercase letters indicate a significant difference (*p* < 0.05) between samples s at different biopolymer ratios.

The particle size and CBD retention rate of nanoparticles with heating at 80 °C were shown in Figure 6. The particle size of zein nanoparticles was relatively constant in the first 30 min, and then significantly increased up to 60 min (*p* < 0.05). The results showed that CBD/zein exhibited a great thermostability when exposed to the thermal process for short time since, the thermal denaturation of zein was about 100 °C [34]. On the other hand, CBD/zein–WP exhibited a significantly increasing trend in 30 min (*p* < 0.05) and kept steady from 60 to 90 min. WP is heat-sensitive, and polymerization occurred under thermal treatment, thus promoting particle size [28]. After heating at 80 °C, the whey protein denatured and aggregated because the denature temperature of whey protein is about 70 °C [35]. This was responsible for the increased particle size in the first 30 min and the unchanged size in the following 60 min, probably because most proteins had already been involved in the unfolding and aggregation [35]. As demonstrated in Figure 6B, the retention rate of CBD in composite nanoparticles was considerably higher than that in zein nanoparticles after thermal treatment at 80 °C for 90 min, suggesting that composite nanoparticles had the remarkable advantage of preventing CBD from thermal-induced degradation. These results may be explained by the fact that the thick core–shell layer of nanoparticle could act as a physical barrier that protected CBD inside [22] which prevented its leakage into water.

Light is a major factor causing the oxidation, isomerization, and oligomerization of CBD [36], which is one of the reasons for its loss of biological activity [37]. Our preliminary study showed that free CBD declined dramatically to 0.44% after UV light treatment for 90 min. This result confirmed that CBD was extremely susceptible to light exposure due to the UV absorption capacity of the aromatic ring in CBD. However, after entrapment in zein–WP composite nanoparticles, the retention rate of CBD was remarkedly enhanced to over 90% and remained relatively stable with small fluctuations during the 90-min irradiation period (Figure 6C). The results testified that encapsulation was effective in preventing the photochemical degradation of CBD. Protein molecules may absorb or block light to delay the photodegradation of bioactive ingredients due to the light absorption of aromatic side groups and double bonds in molecules [38]. Moreover, the formation of the WP layer provided a stronger physical barrier, which hindered the transmission of UV light and the exposure of CBD.

### 3.8. Storage Stability 

**Figure 7:**
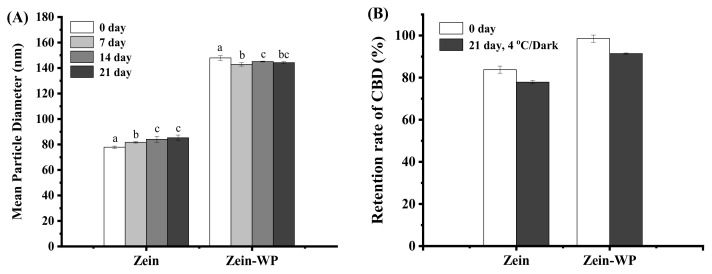
Influence of storage period on mean the particle diameter (**A**) and retention rate of CBD (**B**) of the composite nanoparticles. Note: different lowercase letters indicate the significant difference (*p* < 0.05) between samples s at different biopolymer ratios.

Evaluation of the storage stability of CBD-loaded composite nanoparticles could predict their shelf life in functional foods. Figure 7A showed that the particle size of CBD/zein significantly (*p* < 0.05) increased from 77.90 ± 0.86 to 85.24 ± 2.15 nm, indicating that aggregation happened with time due to the decreased repulsion between particles. The storage period in 4 °C/dark conditions had a slight impact on the particle size of CBD/zein–WP, indicating that strong electrostatic repulsion and steric hindrance may keep the particles maintained a certain distance from each other to prevent the unwanted severe aggregation [27].

The CBD retention rate was also measured and the results are shown in Figure 7B. Free CBD in 20% ethanol was no longer detectable after 21 days of storage, which verified its instability towards harsh environmental circumstances during preservation [37]. After entrapment, 79% and 86% of CBD remained at the end of storage for zein and zein–WP nanoparticles, respectively. CBD/zein–WP fabricated by modified ASP could effectively prevent the degradation of CBD during storage and it indicated a practical contribution to the food industry.

### 3.9. Bioavailability Analysis of CBD

**Figure 8:**
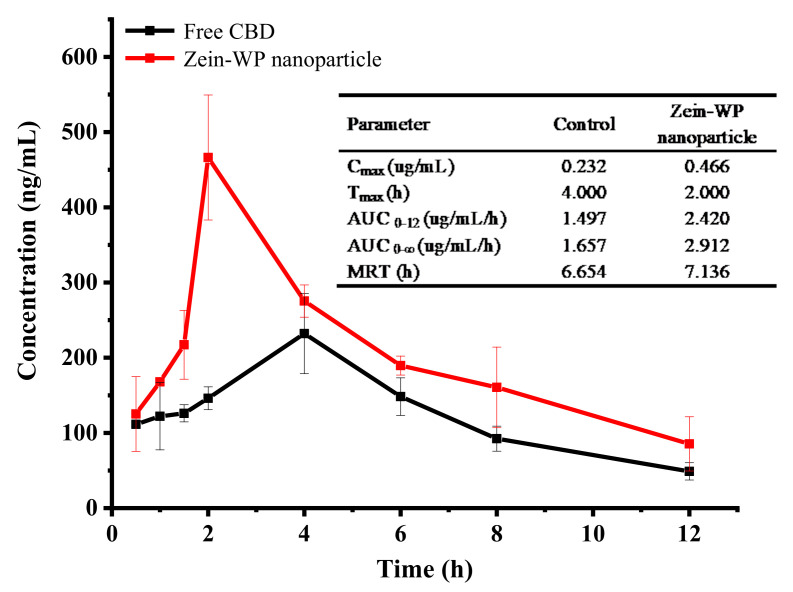
Means of plasma concentration–time profiles and in vivo pharmacokinetic parameters of pure CBD and CBD-loaded zein–WP nanoparticles.

Pharmaceutical kinetic curves of CBD concentration in blood plasma (Figure 8) displayed that CBD concentrations in nanoparticles were remarkably higher than those of pure CBD. The maximum concentration (C_max_) and area under curve (AUC) were 0.232 μg/mL and 1.657 μg/mL/h for pure CBD, respectively, confirming the poor absorption of CBD in vivo [39,40]. However, after encapsulation in zein–WP nanoparticles, the C_max_ and AUC_0–∞_ were increased to 0.466 μg/mL and 2.912 μg/mL·h with about 2-fold and 1.75-fold enhancements, respectively. The results indicated that a high degree of CBD was absorbed into blood circulation in rats after administration. In addition, a lower time to maximum concentration (T_max_) (2 h) occurred with the CBD/zein–WP nanoparticles in comparison to free CBD (4 h), indicating less time was required to reach the maximum concentration after administration with an extensively enhanced intestinal absorption rate [4], which would be preferable for the treatment of seizures associated with Dravet syndrome and Lennox–Gastaut syndrome [41]. CBD in nanoparticles was still detectable in plasma at 12 h, indicating a considerable enhancement in oral bioavailability [42]. 

The enhancement of the bioavailability of CBD in zein–WP nanoparticles may be attributed to the following reasons. First, encapsulation in nanoparticles makes insoluble CBD well dispersed in water, which increases its concentration gradient in the gastrointestinal tract and improves the passive transport of CBD across the epithelium [26,43]. Second, zein and WP are biomaterials with mucoadhesive properties. The loading of CBD in such materials improved its adhesion in the gastrointestinal tract, which further facilitated its absorption in vivo [12]. Third, WP has been reported to be stable in presence of pepsin but is degraded rapidly by pancreatin [44]. The adsorption of WP on the zein core limited enzyme access and protected CBD through to the gastric environment. Fourth, nanoparticles can be taken up by epithelial cells through endocytosis and particle size is the most important physical property in determining the endocytic pathways [12]. The fabricated nanoparticles in this study were at a small size scale of 140–160 nm and were more easily endocytosed [45]. 

## 4. Conclusions

CBD-loaded zein–whey protein composite nanoparticles were successfully fabricated by a modified anti-solvent approach. The size, surface charge, particle size distribution, solubility, encapsulation efficiency, and re-dispersibility of nanoparticles were influenced by the zein to whey protein ratio. A relatively small stable nanoparticle with good dispersity was constructed at ratio of 1:4. The stability of CBD towards harsh environments and storage was remarkably enhanced after entrapment. Pharmacokinetic study showed that CBD in whey protein-coated zein nanoparticles had 2-times and 1.75-fold improvement in maximum concentration (C_max_) and area under curve (AUC) in comparison with free-form CBD. The work would be helpful for improving the application of hydrophobic functional substances in the functional foods and pharmaceutical fields.

## Data Availability

The datasets generated for this study are available on request to the corresponding author.
